# Supplementary material for: PKIS deep dive yields a chemical starting point for dark kinases and a cell active BRSK2 inhibitor
Source: Sci Rep. 2020 Sep 28;10:15826. doi: 10.1038/s41598-020-72869-9 (PMC7522982; doi:10.1038/s41598-020-72869-9)
Supplement: Supplementary file 1 — Supplementary Information [file 41598_2020_72869_MOESM1_ESM.pdf]

Supplementary information

PKIS Deep Dive Yields a Chemical Starting Point for Dark Kinases and a Cell Active BRSK2 Inhibitor

Tigist Y. Tamir[1], David H. Drewry[2, 3], Carrow Wells[2, 3], M. Ben Major[1, 4, 5], *Alison D. Axtman[2, 3]

[1]Department of Pharmacology, University of North Carolina at Chapel Hill, Chapel Hill, NC, USA
[2]Structural Genomics Consortium, UNC Eshelman School of Pharmacy, University of North Carolina at Chapel Hill, Chapel Hill, NC, USA
[3]Division of Chemical Biology and Medicinal Chemistry, UNC Eshelman School of Pharmacy, University of North Carolina at Chapel Hill, NC, USA
[4]Lineberger Comprehensive Cancer Center, University of North Carolina, Chapel Hill, NC, USA
[5]Department of Cell Biology and Physiology, Washington University in St. Louis, St. Louis, MO, USA
**Corresponding author e-mail:* alison.axtman@unc.edu

Supplemental Table 1. Select broad screening results around PKIS compound GW296115.

Kinase	Nanosyn % Inhibition at 1 µM	TSA ΔT_m at 10 µM (°C)	IDG kinase
BRSK1	102	NT	Y
BRSK2	95	NT	Y
RSK3	92	NT	N
STK17B/DRAK2	NT	18.3	Y
PIM3	83	12.0	N
PRKAA2	52[1]	10.6	N
STK33	NT	9.8	Y
PIM2	68	9.8	N
GAK	NT	8.4	N

[1]Protein kinase AMP-activated catalytic subunit alpha 2

Supplemental Table 2. Select DiscoverX and Eurofins data generated for GW296115.

Kinase	DiscoverX % Inhibition at 1 µM	>90%I at Nanosyn or >7.5°C ΔT_m in Table 1	IDG kinase	Assay offered by Eurofins	Eurofins IC_{50} (nM)	Family
STK17B/DRAK2	100	Y (NS)	Y	Y	5.5	CAMK
LRRK2	100	NT	N	Y	25	TKL
MAP3K19	100	NT	N	N	NT	STE
PDGFRB	99.8	NT	N	Y	>10000	TK
STK17A/DRAK1	99.6	NT	Y	Y	28	CAMK
MAP2K5	99.5	NT	N	Y	>10000	STE
PLK4	99.5	NT	N	Y	17	OTHER
PRKG2/PKG2	98.8	NT	N	Y	6.3	AGC
TNIK	97.7	NT	N	Y	10	STE
DAPK3/ZIPK	96.9	NT	N	Y	100	CAMK
GAK	96.7	Y (TSA)	N	N	NT	OTHER
DAPK2	96.7	NT	N	Y	21	CAMK
MINK1/MINK	96	NT	N	Y	35	STE
MYLK/MLCK/smMLCK	95.4	NT	N	Y	49	CAMK
MAP3K9/MLK1	95.1	NT	N	Y	26	TKL
RPS6KA6/RSK4	94.7	NT	N	Y	12	CAMK
PIM3	94.4	Y (NS and TSA)	N	Y	89	CAMK
AURKC	94.1	NT	N	Y	4100	OTHER
CHEK2	92.5	NT	N	Y	2.6	CAMK
MAP3K11/MLK3	92.4	NT	N	Y	1.5	TKL
STK33	92	Y (TSA)	Y	Y	110	CAMK
FLT3	91.5	NT	N	Y	37	TK
PDGFRA	91.4	NT	N	Y	710	TK
MAP4K4/HGK	91.4	NT	N	Y	29	STE
SGK1/SGK	91.2	NT	N	Y	710	AGC
AURKB	90	NT	N	Y	94	OTHER
RIOK3	89	NT	Y	N	NT	ATYPICAL

SGK3	86	NT	N	Y	>10000	AGC
NUAK1/ARK5	85	NT	N	Y	15	CAMK
EPHB6	85	NT	N	N	NT	TK
RPS6KA3/RSK2	84	Y (NS)	N	Y	11	CAMK
BRSK2	83	Y (NS)	Y	Y	8.4	CAMK
DCLK3/DCAMKL3	83	NT	Y	Y	89	CAMK
PIM2	83	Y (NS and TSA)	N	Y	10	CAMK
BRSK1	82	Y (NS)	Y	Y	21	CAMK
PHKG1	82	NT	Y	Y	20	CAMK
MYLK3/caMLCK	80	NT	N	N	NT	CAMK
DAPK1	78	NT	N	Y	>10000	CAMK
ROS1	76	NT	N	Y	>10000	TK
AXL	75	NT	N	Y	160	TK
DMPK	75	NT	N	Y	>10000	AGC

NS: Nanosyn; NT: not tested; TSA: thermal shift assay

Supplemental Table 3. Antibodies.

Antibody	Source	Reference #	Dilution (v:v)
**Mouse anti-FLAG**	Sigma	F3165	1:1000
**Mouse anti-GAPDH**	Sigma	G8795	1:1000
**Mouse anti-Vinculin**	Santa Cruz	sc25336	1:1000
**Rabbit anti-phospho AMPK Substrate Motif [LXRXX(pS/pT)**	Cell Signaling Technologies	5759	1:1000
**Rabbit anti-AMPK**	Cell Signaling Technologies	2532	1:1000
**Rabbit anti-phospho AMPK T172**	Cell Signaling Technologies	2535	1:1000
**Rabbit anti-ULK1**	Cell Signaling Technologies	4776	1:1000
**Rabbit anti-phospho ULK1 S317**	Cell Signaling Technologies	12753	1:1000
**Rabbit anti-phospho ULK1 S757**	Cell Signaling Technologies	6888	1:1000
**Rabbit anti-SQSTM1/P62**	Bethyl	A302-856A	1:1000
**Rabbit anti-phospho SQSTM1/P62 S351**	MBL International	PM074	1:1000

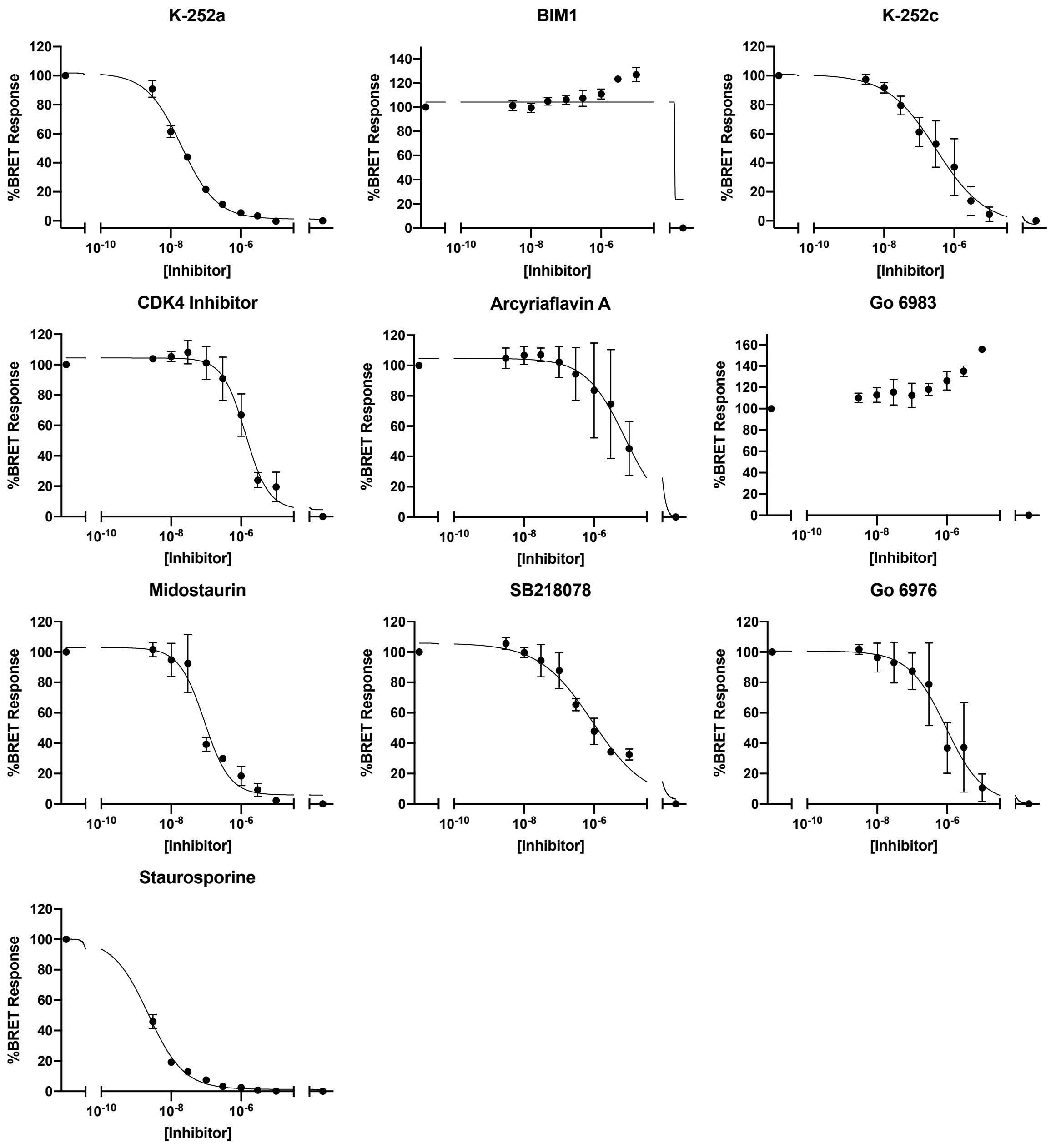

Supplemental Figure 1. NanoBRET cellular target engagement of indolocarbazoles and bisindolylmaleimides shown in **Fig. 5A** and **5B**.

Figure 3B

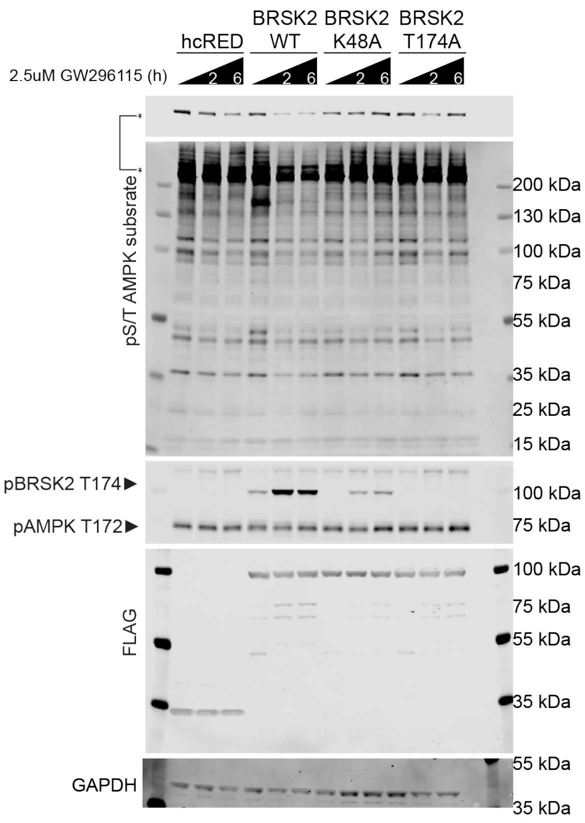

Figure 4A

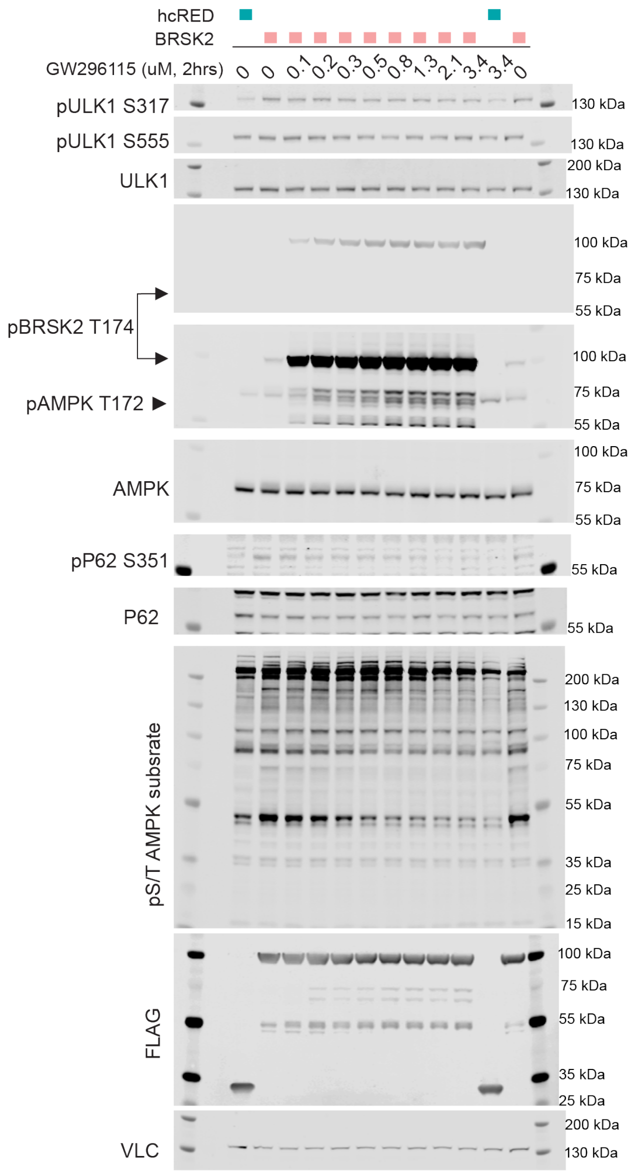

Supplemental Figure 2. Unformatted western blot images corresponding to **Fig. 3B** and **Fig. 4A**.
